# Timing of Lumbar Spinal Fusion Affects Total Hip Arthroplasty Outcomes

**DOI:** 10.5435/JAAOSGlobal-D-19-00133

**Published:** 2019-11-04

**Authors:** Abiram Bala, Deepak V. Chona, Derek F. Amanatullah, Serena S. Hu, Kirkham B. Wood, Todd F. Alamin, Ivan Cheng

**Affiliations:** From the Department of Orthopaedic Surgery, Stanford University Medical Center, Stanford, CA.

## Abstract

Many patients are affected by concurrent disease of the hip and spine, undergoing both total hip arthroplasty (THA) and lumbar spinal fusion (LSF). Recent literature demonstrates increased prosthetic dislocation rates in patients with THA done after LSF. Evidence is lacking on which surgery to do first to minimize complications. The purpose of this study was to evaluate the effect of timing between the two procedures on postoperative outcomes.

**Methods:**

We queried the Medicare standard analytics files between 2005 and 2014. Four groups were identified and matched by age and sex: THA with previous LSF, LSF with previous THA, THA with spine pathology without fusion, and THA without spine pathology. Revision THA or LSF and bilateral THA were excluded. Comorbidities and Charlson Comorbidity Index were identified. Postoperative complications at 90 days and 2 years were calculated after the most recent surgery. Four-way chi-squared and standard descriptive statistics were calculated.

**Results:**

Thirteen thousand one hundred two patients had THA after LSF, 10,482 patients had LSF after THA, 104,820 had THA with spine pathology, and 492,654 had THA without spine pathology. There was no difference in the Charlson Comorbidity Index score between the THA after LSF and LSF after THA groups. There was a statistically significant difference in THA dislocation rate, with LSF after THA at 1.7%, THA without spine pathology at 2.3%, THA with spine pathology at 3.3%, and THA after LSF at 4.6%. There was a statistically significant difference in THA revision rate, with THA without spine pathology at 3.3%, LSF after THA at 3.7%, THA with spine pathology at 4.2%, and THA after LSF at 5.7%.

**Conclusion:**

LSF after THA is associated with a reduced dislocation rate compared with THA after LSF. Reasons may include decreasing pelvic mobility in a stable, well-healed THA or early postoperative spine precautions after LSF restricting positions of dislocation.

Hip-spine syndrome, the concurrent existence of degenerative conditions of both the hip joint and the spine, can have overlapping symptoms making specific identification and treatment of pathology difficult.^1,2^ Both total hip arthroplasty (THA) and lumbar spinal fusion (LSF) done for degenerative disease can provide pain relief and improve functional outcomes in many patients, and, correspondingly, the utilization of both procedures continues to increase rapidly.^3,4^

When switching from the standing to the sitting position, a balanced and flexible spine allows for pelvic retroversion, which leads to increased functional anteversion of the acetabulum.^5-7^ This allows for adequate coverage of the femoral head in the sitting position as the hip goes into flexion. Degenerative conditions of the lumbar spine can lead to a stiff spine which limits the mobility necessary for changes in functional version of the acetabulum. This can put a patient at risk of intra-articular impingement and instability.^7,8,9,10^ Approximately two percent of Medicare patients who undergo THA have had previous LSF, with between 18% and 25% having seen a spine surgeon before proceeding with arthroplasty.^11,12,13,14^ With spinal deformity, degenerative disease, and LSF now known as risk factors for alteration of functional acetabular position, even with cup placement in the traditional “safe zone,” it is no surprise that there are many emerging data demonstrating higher dislocation rates of THA after previous spinal fusion.^9,11,15,16^

A recent meta-analysis by An et al. found that previous LSF increases the relative risk of THA dislocation two-fold.^17^ As such, it is a known complication that surgeons should routinely counsel patients who present for evaluation of primary THA in the setting of previous LSF. What remains unclear in both practice and in the literature, however, is the appropriate sequence by which to treat patients who have concurrent surgical hip and spine pathology to minimize these risks. The purpose of this study was to evaluate whether timing of LSF, either before or after THA, affects prosthetic dislocation and other postoperative complications.

## Methods

The study used completely deidentified patient information and was exempt from Institutional Review Board approval. We queried the entire Medicare database from 2005 to 2014 containing 100% of administrative records on more than 51 million patients using Pearl Diver technologies. First, we identified all patients who had THA done using *International Classification of Diseases-9* procedure code 81.51 and Current Procedural Terminology code 27130. Next, we selected for patients who only had a single primary THA done and with a minimum of 2-year follow-up per patient after surgery.

We then identified all patients who had primary LSF done using the relevant *International Classification of Diseases-9* and Current Procedural Terminology codes (a full list of codes used in this study is available in the Appendix 1). We excluded all patients who had revision LSF. Each patient was selected to have only a single episode of LSF as well as a minimum of 2 years of follow-up. Next, we identified a cohort of patients who had known lumbar spine pathology, however, who did not undergo either primary or revision LSF at any point on their record.^18^

With these cohorts identified, we created four groups for comparison. Group one included patients who had THA done after LSF. Group two included patients who had LSF done after THA. Group three included patients who had known lumbar spine pathology who underwent THA, never having LSF at any time point in their record. Group four, our true control, was the group of patients who underwent THA without any LSF or diagnosis of spine pathology. Each group had a minimum of 2 years of follow-up from the latest procedure. Using a stepwise algorithm, all four study groups were matched by age and sex.

Surgical complications were assessed at both 90 days and 2 years. To prevent confounding from codes previously on patients' records, the “first_instance” command was used on all complications of interest, allowing us to identify the first time the complication occurred to prevent any pre-existing diagnoses from skewing the results. Complications were tracked from the latest procedure. Demographics were identified, comorbidities were found using the standardized Elixhauser measure, and the Charlson comorbidity index (CCI) for each group was calculated.^19,20^ Four-way chi-squared and associated *P* values were calculated to compare all four groups at once. Additional subgroup analysis with standard descriptive statistics was done between the THA after LSF and the LSF after THA groups. Significance was set at an alpha of <0.05.

We identified and matched 13,102 patients who had THA after LSF (group 1), 10,482 patients who had LSF after THA (group 2), 104,820 patients with spine pathology without LSF who underwent THA (group 3), and 492,654 patients who had THA done without any spine pathology or LSF (group 4). With the use of the matching system, there were no differences in age and sex proportions among the four groups (Table 1).

**Table 1:** Demographics

Factors	THA After LSF	LSF After THA	THA, Spine Path, No LSF	THA, No Spine Path	*P* Value
n	13,102	10,482	104,820	492,654	
Age					1.000
64 and younger	1497 (11%)	1198 (11%)	11,980 (11%)	56,304 (11%)	
65-69	2531 (19%)	2025 (19%)	20,250 (19%)	95,173 (19%)	
70-74	3699 (28%)	2959 (28%)	29,589 (28%)	139,072 (28%)	
75-79	3210 (25%)	2568 (24%)	25,680 (24%)	120,694 (24%)	
80-84	1662 (13%)	1330 (13%)	13,300 (13%)	62,508 (13%)	
85 and over	503 (4%)	402 (4%)	4019 (4%)	18,894 (4%)	
Sex					1.000
Female	8096 (62%)	6477 (62%)	64,768 (62%)	304,415 (62%)	
Male	5006 (38%)	4005 (38%)	40,050 (38%)	188,230 (38%)	
2005	247 (2%)	101 (1%)	3628 (3%)	77,554 (16%)	
2006	804 (6%)	506 (5%)	8599 (8%)	66,202 (13%)	
2007	1161 (9%)	797 (8%)	11,175 (11%)	62,732 (13%)	
2008	1345 (10%)	1062 (10%)	11,524 (11%)	53,494 (11%)	
2009	1773 (14%)	1565 (15%)	14,715 (14%)	58,402 (12%)	
2010	2242 (17%)	1810 (17%)	17,038 (16%)	58,144 (12%)	
2011	2515 (19%)	2138 (20%)	18,002 (17%)	57,044 (12%)	
2012	3015 (23%)	2503 (24%)	20,137 (19%)	59,073 (12%)	

LSF = lumbar spinal fusion, THA, total hip arthroplasty

## Results

There were differences in Elixhauser medical comorbidities (*P* < 0.001), with those having THA without spine pathology with the lowest proportions, indicating the healthiest cohort (Table 2). Using a four-way analysis of variance test, we found differences in the CCI score (*P* < 0.001), with THA after LSF at 5.3 (SD 2.1), LSF after THA at 5.4 (SD 2.2), THA with spine pathology without LSF at 5.4 (SD 2.2), and THA without spine pathology at 4.8 (SD 1.9). However, when specifically comparing our two main groups of interest, THA after LSF and LSF after THA, a *t*-test demonstrated no difference in the CCI score (*P* = 0.287), indicating similar health status.

**Table 2:** Comorbidities

Factors	THA After LSF	LSF After THA	THA, Spine Path, No LSF	THA, No Spine Path	*P* Value
n	13,102	10,482	104,820	492,654	
Comorbidity					
Congestive heart failure	1562 (12%)	1288 (12%)	13,268 (13%)	35,493 (7%)	<0.001
Valvular disease	1969 (15%)	1595 (15%)	15,404 (15%)	38,374 (8%)	<0.001
Pulmonary circulation disorders	727 (6%)	602 (6%)	5644 (5%)	13,094 (3%)	<0.001
Peripheral vascular disease	2763 (21%)	2193 (21%)	22,218 (21%)	42,816 (9%)	<0.001
HTN (uncomplicated)	10,945 (84%)	8941 (85%)	78,231 (75%)	245,620 (50%)	<0.001
HTN (complicated)	1797 (14%)	1597 (15%)	13,816 (13%)	33,723 (7%)	<0.001
HTN (uncomplicated and complicated)	10,999 (84%)	8991 (86%)	78,881 (75%)	250,089 (51%)	<0.001
Paralysis	480 (4%)	217 (2%)	1995 (2%)	4928 (1%)	<0.001
Other neurological disorders	1662 (13%)	1419 (14%)	12,326 (12%)	27,047 (5%)	<0.001
Chronic pulmonary disease	4196 (32%)	3433 (33%)	31,290 (30%)	77,702 (16%)	<0.001
Diabetes without chronic complications	3796 (29%)	3101 (30%)	27,491 (26%)	79,674 (16%)	<0.001
Diabetes with chronic complications	886 (7%)	770 (7%)	6639 (6%)	14,747 (3%)	<0.001
Hypothyroidism	3501 (27%)	2857 (27%)	24,600 (23%)	67,746 (14%)	<0.001
Renal failure	1307 (10%)	1109 (11%)	9721 (9%)	25,912 (5%)	<0.001
Liver disease	520 (4%)	444 (4%)	4848 (5%)	9497 (2%)	<0.001
Chronic peptic ulcer disease	47 (0%)	24 (0%)	301 (0%)	648 (0%)	<0.001
HIV/AIDS	21 (0%)	17 (0%)	353 (0%)	1309 (0%)	<0.001
Lymphoma	187 (1%)	149 (1%)	1814 (2%)	4627 (1%)	<0.001
Metastatic cancer	196 (1%)	169 (2%)	2353 (2%)	6071 (1%)	<0.001
Solid tumor without metastasis	1518 (12%)	1258 (12%)	15,169 (14%)	44,233 (9%)	<0.001
Rheumatoid arthritis/collagen vascular diseases	2046 (16%)	1730 (17%)	14,491 (14%)	28,413 (6%)	<0.001
Coagulation deficiency	1038 (8%)	978 (9%)	6457 (6%)	17,073 (3%)	<0.001
Obesity	2850 (22%)	2439 (23%)	16,189 (15%)	34,765 (7%)	<0.001
Weight loss	998 (8%)	704 (7%)	7947 (8%)	17,727 (4%)	<0.001
Fluid and electrolyte disorders	4317 (33%)	3435 (33%)	26,827 (26%)	69,409 (14%)	<0.001
Blood loss anemia	614 (5%)	580 (6%)	3014 (3%)	8305 (2%)	<0.001
Deficiency anemias	4896 (37%)	4606 (44%)	29,178 (28%)	79,446 (16%)	<0.001
Alcohol abuse	392 (3%)	365 (3%)	3167 (3%)	8606 (2%)	<0.001
Drug abuse	469 (4%)	332 (3%)	3162 (3%)	5168 (1%)	<0.001
Psychoses	977 (7%)	756 (7%)	7544 (7%)	15,884 (3%)	<0.001
Depression	3178 (24%)	2739 (26%)	19,354 (18%)	38,734 (8%)	<0.001
Average CCI score	5.33	5.36	5.43	4.75	<0.001
Median CCI score	5	5	5	4	
SD for CCI score	2.14	2.17	2.32	1.93	

CCI = Charlson Comorbidity Index, HTN = hypertension, LSF = lumbar spinal fusion, THA = total hip arthroplasty

THA after LSF had the highest rate of dislocation at 90 days (2.8%), followed by THA with spine pathology (1.9%) and THA without spine pathology (1.2%). LSF after THA had the lowest 90-day dislocation rate of 0.2% (*P* < 0.001) (Table 3). The odds of an early dislocation were increased by 16.6-fold (95% confidence interval [CI] 10.3 to 26.7, *P* < 0.001) when THA was done after LSF in comparison with LSF done after THA. That is an absolute risk reduction of 2.6% in the rate of early dislocation if LSF is done after THA rather than before THA. Hence, doing LSF after THA in 39 cases prevents one early dislocation caused by THA after LSF. THA revision rates at 90 days followed a similar trend, with THA after LSF at 2.0%, THA with spine pathology at 1.8%, THA without spine pathology at 1.4%, and LSF after THA at 0.2% (*P* < 0.001). The odds of an early revision were increased by 10.3-fold (95% CI, 6.6 to 16.5, *P* < 0.001) when THA was done after LSF in comparison with LSF done after THA. That is an absolute risk reduction of 1.8% in the rate of early THA revision if LSF is done after THA rather than before THA. Hence, doing LSF after THA in 56 cases prevents one early THA revision caused by THA after LSF.

**Table 3:** 90-Day Surgical Complication Rates

Factors	THA After LSF	LSF After THA	THA, Spine Path, No LSF	THA, No Spine Path	4-Way X^2^ *P*	THA After LSF Versus LSF After THA Only (OR, 95% CI)	*P* Value
n	13,102	10,482	104,820	492,654			
Complication							
Wound complication	64 (0.5%)	136 (1.3%)	429 (0.4%)	1362 (0.3%)	<0.001	0.373 (0.277-0.503)	<0.001
Vascular injury	26 (0.2%)	17 (0.2%)	216 (0.2%)	1051 (0.2%)	0.675	1.224 (0.664-2.257)	0.517
PJI	157 (1.2%)	14 (0.1%)	1217 (1.2%)	4071 (0.8%)	<0.001	9.068 (5.246-15.675)	<0.001
Periprosthetic fracture	111 (0.8%)		684 (0.7%)	2413 (0.5%)			
Implant dislocation	365 (2.8%)	18 (0.2%)	1947 (1.9%)	6136 (1.2%)	<0.001	16.659 (10.371-26.760)	<0.001
Implant loosening	39 (0.3%)	18 (0.2%)	256 (0.2%)	873 (0.2%)	<0.001	1.736 (0.992-3.036)	0.050
Broken implant	18 (0.1%)		200 (0.2%)	746 (0.2%)			
Cellulitis	231 (1.8%)	65 (0.6%)	2033 (1.9%)	7284 (1.5%)	<0.001	2.876 (2.182-3.792)	<0.001
Other postoperative infection	203 (1.5%)	359 (3.4%)	1822 (1.7%)	6735 (1.4%)	<0.001	0.444 (0.373-0.528)	<0.001
Heterotopic ossification	13 (0.1%)		141 (0.1%)	439 (0.1%)			
Cup-liner dissociation	38 (0.3%)		324 (0.3%)	1028 (0.2%)			
THA revision	266 (2.0%)	21 (0.2%)	1854 (1.8%)	6822 (1.4%)	<0.001	10.323 (6.615-16.109)	<0.001
THA arthrotomy/I&D	119 (0.9%)	16 (0.2%)	961 (0.9%)	3466 (0.7%)	<0.001	5.996 (3.555-10.110)	<0.001

CI = confidence interval, I&D = irrigation and débridement, LSF = lumbar spinal fusion, OR = odds ratio, PJI = periprosthetic joint infection, THA = total hip arthroplasty

Blank cells indicate cases with insufficient data for analysis.

THA after LSF had the highest rate of dislocation at 2 years (4.6%), followed by THA with spine pathology (3.2%) and THA without spine pathology (2.3%). LSF after THA had the lowest 2-year dislocation rate of 1.7% (*P* < 0.001) (Table 4). The odds of a late dislocation were increased by 2.8-fold (95% CI, 2.4 to 3.4, *P* < 0.001) when THA was done after LSF in comparison with LSF done after THA. That is an absolute risk reduction of 2.9% in the rate of late dislocation if LSF is done after THA rather than before THA. Hence, doing LSF after THA in 35 cases prevents one late dislocation caused by THA after LSF. THA revision rates at 2 years followed a similar trend, with THA after LSF at 5.7%, THA with spine pathology at 4.2%, THA without spine pathology at 3.3%, and LSF after THA at 3.7% (*P* < 0.001). The odds of a late revision were increased by 1.6-fold (95% CI, 1.4 to 1.8, *P* < 0.001) when THA was done after LSF in comparison with LSF done after THA. That is an absolute risk reduction of 2.0% in the rate of late THA revision if LSF is done after THA rather than before THA. Hence, doing LSF after THA in 50 cases prevents one late THA revision caused by THA after LSF.

**Table 4:** 2-Year Surgical Complication Rates

Factors	THA After LSF	LSF After THA	THA, Spine Path, No LSF	THA, No Spine Path	4-Way X^2^ *P*	THA After LSF Versus LSF After THA Only (OR, 95% CI)	*P* value
n	13,102	10,482	104,820	492,654			
Complication							
Wound complication	132 (1.0%)	214 (2.0%)	909 (0.9%)	2988 (0.6%)	<0.001	0.488 (0.392-0.608)	<0.001
Vascular injury	49 (0.4%)	41 (0.4%)	386 (0.4%)	1703 (0.3%)	0.578	0.956 (0.631-1.449)	0.832
PJI	352 (2.7%)	174 (1.7%)	2470 (2.4%)	8531 (1.7%)	<0.001	1.636 (1.361-1.965)	<0.001
Periprosthetic fracture	179 (1.4%)	52 (0.5%)	1174 (1.1%)	4236 (0.9%)	<0.001	2.778 (2.038-3.787)	<0.001
Implant dislocation	608 (4.6%)	174 (1.7%)	3329 (3.2%)	11,229 (2.3%)	<0.001	2.883 (2.431-3.419)	<0.001
Bearing surface wear	25 (0.2%)	26 (0.2%)	141 (0.1%)	573 (0.1%)	<0.001	0.769 (0.444-1.332)	0.347
Osteolysis	22 (0.2%)	29 (0.3%)	98 (0.1%)	517 (0.1%)	<0.001	0.606 (0.348-1.056)	0.074
Implant loosening	220 (1.7%)	200 (1.9%)	1267 (1.2%)	4243 (0.9%)	<0.001	0.878 (0.724-1.065)	0.187
Broken implant	70 (0.5%)	50 (0.5%)	526 (0.5%)	2102 (0.4%)	0.003	1.121 (0.779-1.612)	0.539
Cellulitis	680 (5.2%)	383 (3.7%)	5528 (5.3%)	19,844 (4.0%)	<0.001	1.443 (1.270-1.640)	<0.001
Other postoperative infection	431 (3.3%)	553 (5.3%)	3308 (3.2%)	12,153 (2.5%)	<0.001	0.611 (0.537-0.695)	<0.001
Heterotopic ossification	33 (0.3%)	15 (0.1%)	414 (0.4%)	1014 (0.2%)	<0.001	1.762 (0.957-3.246)	0.066
Cup-liner dissociation	221 (1.7%)	151 (1.4%)	1167 (1.1%)	3653 (0.7%)	<0.001	1.174 (0.953-1.446)	0.132
THA revision	745 (5.7%)	384 (3.7%)	4387 (4.2%)	16,198 (3.3%)	<0.001	1.585 (1.398-1.798)	<0.001
THA arthrotomy/I&D	207 (1.6%)	106 (1.0%)	1757 (1.7%)	6232 (1.3%)	<0.001	1.571 (1.242-1.989)	<0.001

CI = confidence interval, I&D = irrigation and débridement, LSF = lumbar spinal fusion, OR = odds ratio, PJI = periprosthetic joint infection, THA = total hip arthroplasty

References

## Discussion

As the utilization of THA and LSF increases and the general population ages, the cohort of patients who present with concurrent degenerative hip and spine disease who benefit from both interventions will also increase.^1^ As our understanding of spinopelvic mobility and its effect on the mechanics of the hip joint continues to improve, so has the literature solidifying both previous LSF and degenerative lumbar disease as risk factors for THA dislocation.^7,17,18^ We sought to answer the question of which surgery should be done first to minimize complications. Our data suggest that LSF should be done after THA to minimize dislocation and revision risk.

Our results of increased dislocation, revision, and overall surgical complications for THA done after LSF align with the established literature. Buckland et al^16^ found dislocation rates for THA after LSF between 3% and 4%, similar to those seen in our study. Sing et al found 2-year revision rates for THA after LSF between 5% and 7%, again reflecting our findings.^11^ In addition, our data also corroborate the literature which demonstrates that lumbar spine disease in the absence of LSF also places patients at risk of dislocation and revision after THA.^17,18^ As there are no similar studies examining LSF after THA, we cannot directly compare our results for this group.

Decreased dislocation risk with LSF done after THA in comparison with THA after LSF can be explained from multiple angles. From a biomechanical perspective, a patient who has LSF done after THA may have a stable well-performing THA with a well-positioned acetabular implant in an already stiff and immobile spine. Therefore, the subsequent correction of lumbar lordosis with added stability and stiffness after LSF is insufficient to alter functional anteversion, and the risk of dislocation does not substantially increase.^21^ From a patient selection perspective, patients who have chronic THA instability may not be considered safe surgical candidates, and surgeons may inherently select against doing LSF in patients with a known history of recurrent surgical complications. Although literature to support this explanation is lacking, careful preoperative planning before spine fusion in a value-based health care environment is becoming increasingly important, and thus, such exclusion criteria may be relevant.^22^ Finally, early postoperative spine restrictions limiting bending, lifting, and twisting in the setting of newly altered spinopelvic mobility may prevent patients from engaging in positions predisposing to THA dislocation. This concept, however, requires additional validation.

The capsule, short external rotators, and abductors all contribute to THA stability.^22^ Hip instability is multifactorial with patients falling into early (<3 months) and late (>3 months) instability.^23-25^ Early dislocation is caused by surgical issues related to soft-tissue tension and component position. Late dislocation is due to deteriorating neuromuscular function, polyethylene wear, component migration, or infection.^23^ In this case, a patient with a stable well-performing THA which has had sufficient time for bone ingrowth (if cementless), soft-tissue healing and scarring, and adequate time for surrounding muscular strengthening may be better suited to handle the changes in spinopelvic mobility imposed by an LSF. This is in contrast to having THA done after pre-existing LSF, in which case a new hip prosthesis is placed into an unfavorable mobility environment and does not have a sufficiently healed soft-tissue envelope to protect against instability.

This idea fits conceptually with the timing of the dislocations identified in our study, where THA after LSF had a comparatively higher proportion of early dislocations (2.8% by 90 days and 4.6% by 2 years, a 2-fold increase), while most of the dislocations identified for the LSF after THA group were late (0.2% by 90 days and 1.7% by 2 years, an 8.5-fold increase). Although there is evidence that early soft-tissue healing is seen after THA, it can take multiple years until complete scar maturity.^26^ During this interval, patients with a new primary THA with a previous LSF may be at increased risk until sufficient capsule healing, scar formation, and muscle strengthening are achieved. This concept is not well studied in the THA literature; however, it is observed with modern radial head replacements. In this case, soft-tissue healing and scar formation around the radiocapitellar joint, rather than the mechanical properties and position of the implant, help the elbow joint achieve stability and account for the excellent functional outcomes of nonpress fit, cementless radial head replacements.^27^

Studies involving administrative claims have inherent limitations which are important to discuss in this study. Evidence in both the arthroplasty and spine literature is mixed on the accuracy and validity of administrate claims in identifying postoperative complications.^28,29,30,31^ Moreover, we do not have patient-specific identifiers or direct clinical information that allows for more complex multivariate regression analysis. Although we were able to match patients by age and sex, there were still differences in comorbidities among the groups which may confound our surgical outcomes. However, the fact that our two main groups of interest (THA after LSF, and LSF after THA) had no difference in the CCI score, and a very similar Elixhauser comorbidity profile is reassuring that our question of timing of fusion versus arthroplasty is not confounded by differences in baseline health status. We were also not able to examine surgical approach for the THA as this is not coded in the database, which may have an effect on dislocation based on spinopelvic position. Finally, we were not able to specifically delineate the amount of correction nor specific involvement of sacroiliac fusion, which may also affect our results. However, we hope that our results serve as a springboard for future study in more specific groups which may be able to answer these questions.

As the utilization of both THA and LSF increase, there will be a larger cohort of patients who will benefit from both interventions. Our results suggest that for patients indicated to undergo both procedures, doing THA first with LSF to follow at a later date is associated with a lower risk of instability and revision THA. Reasons may include minimal adjustment in spinopelvic parameters, patient selection, and improved stability from a well-healed soft-tissue envelope. Additional investigation is certainly warranted; yet, we believe these results will help surgeons in counseling patients and in preoperative planning.

## Appendix

**Appendix 1:** List of *ICD-9* and CPT codes used in this study

THA	CPT: 27130, *ICD-9*: 81.51
LSF	*ICD-9*: 81.06-81.08, 81.62, 81.63
LSF revision	*ICD-9*: 813-813.9, CPT: 22849, 22850, 22852, 22855
Wound complication	*ICD-9*: 998.32
Neural deficit	*ICD-9*: 997.00, 965.0-956.9
Vascular injury	*ICD-9*: 997.2
PJI	*ICD-9*: 996.66
Periprosthetic fracture	*ICD-9*: 996.44
Implant dislocation	*ICD-9*: 996.42
Bearing surface wear	*ICD-9*: 996.46
Osteolysis	*ICD-9*: 996.45
Implant loosening	*ICD-9*: 996.41
Broken implant	*ICD-9*: 996.43
Cellulitis	*ICD-9*: 682.6, 682.9
Other postoperative infection	*ICD-9*: 998.59
Heterotopic ossification	*ICD-9*: 728.13
Cup-liner dissociation	*ICD-9*: 996.47
THA revision	CPT: 27090, 27091, 27134, 27137, 27138, *ICD-9*: 81.53, 00.70, 00.71, 00.72,00.73
THA arthrotomy/I&D	*ICD-9*: 80.00, 80.05, 80.10, 80.15, 80.75,CPT: 26990, 26992, 27030, 27033, 27052, 27054, 10140, 27036, 27301, 27303
